# Reduction of respiratory motion artifacts in gadoxetate-enhanced MR with a deep learning–based filter using convolutional neural network

**DOI:** 10.1007/s00330-020-07006-1

**Published:** 2020-06-17

**Authors:** M.-L. Kromrey, D. Tamada, H. Johno, S. Funayama, N. Nagata, S. Ichikawa, J.-P. Kühn, H. Onishi, U. Motosugi

**Affiliations:** 1 Department of Radiology, University of Yamanashi, 1110 Shimokato, Chuo, Yamanashi 409-3898 Japan; 2 Department of Diagnostic Radiology and Neuroradiology, University Medicine Greifswald, Greifswald, Germany; 3 Institute of Diagnostic and Interventional Radiology, University Medicine, Carl-Gustav Carus University, Dresden, Germany

**Keywords:** Machine learning, Artifacts, Magnetic resonance imaging, Gadolinium DTPA

## Abstract

**Objectives:**

To reveal the utility of motion artifact reduction with convolutional neural network (MARC) in gadoxetate disodium–enhanced multi-arterial phase MRI of the liver.

**Methods:**

This retrospective study included 192 patients (131 men, 68.7 ± 10.3 years) receiving gadoxetate disodium–enhanced liver MRI in 2017. Datasets were submitted to a newly developed filter (MARC), consisting of 7 convolutional layers, and trained on 14,190 cropped images generated from abdominal MR images. Motion artifact for training was simulated by adding periodic *k*-space domain noise to the images. Original and filtered images of pre-contrast and 6 arterial phases (7 image sets per patient resulting in 1344 sets in total) were evaluated regarding motion artifacts on a 4-point scale. Lesion conspicuity in original and filtered images was ranked by side-by-side comparison.

**Results:**

Of the 1344 original image sets, motion artifact score was 2 in 597, 3 in 165, and 4 in 54 sets. MARC significantly improved image quality over all phases showing an average motion artifact score of 1.97 ± 0.72 compared to 2.53 ± 0.71 in original MR images (*p* < 0.001). MARC improved motion scores from 2 to 1 in 177/596 (29.65%), from 3 to 2 in 119/165 (72.12%), and from 4 to 3 in 34/54 sets (62.96%). Lesion conspicuity was significantly improved (*p* < 0.001) without removing anatomical details.

**Conclusions:**

Motion artifacts and lesion conspicuity of gadoxetate disodium–enhanced arterial phase liver MRI were significantly improved by the MARC filter, especially in cases with substantial artifacts. This method can be of high clinical value in subjects with failing breath-hold in the scan.

**Key Points:**

*• This study presents a newly developed deep learning–based filter for artifact reduction using convolutional neural network (motion artifact reduction with convolutional neural network, MARC).*

*• MARC significantly improved MR image quality after gadoxetate disodium administration by reducing motion artifacts, especially in cases with severely degraded images.*

*• Postprocessing with MARC led to better lesion conspicuity without removing anatomical details.*

## Introduction

Recent research demonstrated intravenous bolus injection of gadolinium-based contrast agents to be accompanied by motion-related image degradation in the arterial phase [1–3]. This phenomenon is temporary and self-limited, wherefore the term “transient severe motion” (TSM) is often used. Although the underlying pathomechanism has not yet been conclusively solved, the incidence of these artifacts, ranging from 8 to 20% [1, 2, 4, 5], was found to be significantly higher after gadoxetate disodium administration compared to other contrast media [1, 2, 4]. TSM artifacts are of high clinical significance, as the arterial phase is essential for lesion characterization [6, 7]. Artifact reduction during image acquisition or afterwards, therefore, would contribute to the imaging-based diagnosis in the clinical setting.

In order to solve this problem, several strategies are conceivable. One potential solution can be patient-based: through informing patients concerning the problem of acute transient dyspnea or performing breath-hold practice before the scan, the rate of artifacts could be decreased [8]. Likewise, dilution of the contrast agent is able to reduce the incidence of artifacts [9, 10]. Another strategy can be a modulation of the data acquisition: fast scanning techniques leading to a shorter examination time consequently minimize the risk of patient motion. Compressed sensing is now widely used in clinical practice to shorten the acquisition time by the use of undersampled *k*-space data without compromising signal-to-noise ratio [11, 12]. Other studies focused on respiratory triggering, where arterial phase images are acquired with the help of respiratory tracings or navigator echoes [13]. The use of single-breath-hold multi-arterial phase acquisition provides adequate well-timed late hepatic arterial phase images in most patients even with transient severe motion [2, 14, 15]. Interestingly, Min et al [16] showed a similar incidence of TSM artifacts with multiple arterial phases using view sharing from two different vendors and conventional single arterial phase in a retrospective study.

All these approaches, however, have to be prepared before the scan at a time when we do not know if the individual patient will fail their breath-hold, and therefore turn out time-consuming and disrupt the clinical workflow. Postprocessing artifact removal allows for standardized MR protocols and does not require any change in sequence acquisition. On this field, deep learning methods show emerging applications in medical image reconstruction and related reduction of artifacts. In fact, a variety of studies have been published on the field of reconstruction [17–19]. However, in terms of artifact reduction in MRI, there are only a few published papers addressing motion artifacts [20–22]. Especially, the clinical utility of respiratory motion artifact reduction has not been studied so far.

Therefore, the purpose of our study was to reveal the utility of respiratory motion artifact reduction with convolutional neural network (MARC) in combination with advanced acquisition method of single-breath-hold multi-arterial phase acquisition (DISCO) in gadoxetate disodium–enhanced MRI of the liver.

## Materials and methods

The retrospective study was approved by the local institutional review board. The requirement to obtain patients’ informed consent was waived.

A study coordinator searched the electronic database for abdominal MR examinations under administration of gadoxetate disodium and found 192 consecutive examinations between March and June 2017. No examination had to be excluded. The study population consisted of 131 men and 61 women with a mean age of 68.7 ± 10.3 years (range 29–89 years).

### Magnetic resonance imaging acquisition

MR examinations were performed using a 3-T system (MR 750, GE Healthcare) with a 32-channel torso phased-array coil. Pre-contrast and 6 arterial dynamic phases were acquired each during breath-hold. Gadoxetate disodium (EOB Primovist^®^, Bayer-Schering Pharma) was administered intravenously at a rate of 1 ml/s in a standard dose of 0.025 mmol/kg body weight with following saline flush.

Multiphasic hepatic arterial phase imaging was performed using multiphasic T1-weighted 3D spoiled gradient-echo sequence with view sharing technique (differential subsampling with Cartesian ordering, DISCO), using the following parameters: TR = 3.9 ms, TE = 1.1/2.2 ms, flip angle = 15°, receiver bandwidth = ± 167 kHz, field of view = 340 × 340 mm^2^, slice thickness/intersection gap = 3.6/0 mm, matrix = 320 × 192, parallel imaging factor = phase 2.0/slice 1.5, acquisition time = 22–26 s, and temporal resolution = ~ 4 s.

### Image analysis

For the whole study population of 192 patients, one pre-contrast phase and 6 dynamic post-contrast arterial phases per patient resulted in a total of 1344 image sets for original and artifact-reduced data. Image analyses of T1-weighted sequences were performed independently by two observers with 3 and 11 years of experience in abdominal MR imaging, who were blinded to non-imaging-based patient risk factors, as well as whether images were the originals or artifact-reduced, that is after the MARC filter application.

**Fig. 1:**
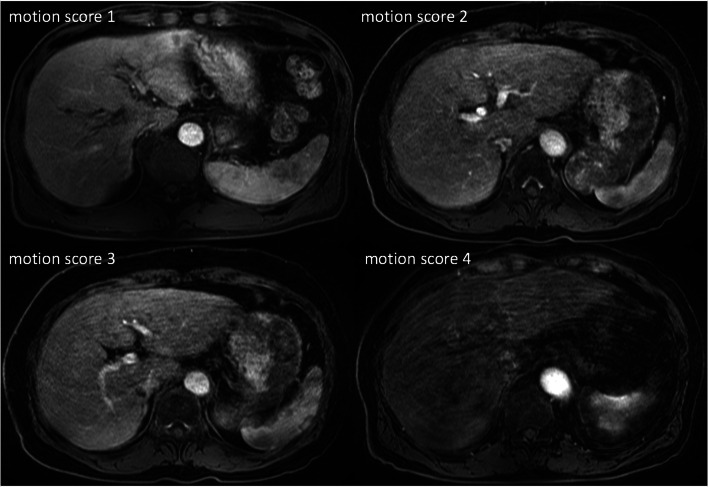
Ranking of motion artifacts in MR imaging on a 4-point scale. Axial T1-weighted transverse MR images following intravenous gadoxetate disodium application. Motion scores were categorized as 1 (no artifact), 2 (mild artifact, no effect on diagnostic quality), 3 (moderate artifact, impeded diagnostic quality), and 4 (severe artifact, non-diagnostic)

Image quality was evaluated in terms of motion artifacts and diagnostic validity using a 4-point scoring system: 1 = no motion artifacts, 2 = minor motion artifacts/no effect on diagnostic quality, 3 = distinct artifacts/impeded diagnostic quality, and 4 = severe artifacts/non-diagnostic image quality. Figure 1 shows examples of the different motion scores in the arterial phase of MR imaging.

Additionally, a radiologist reviewed all original and T2-weighted images to find a focal liver lesion per patient with referring to the radiological reports. If more than one lesion was present in the liver, selection was performed in the following order: (1) hepatocellular carcinoma (HCC), (2) malignant tumor, (3) benign tumor (i.e., hemangioma), (4) benign non-tumorous lesion (i.e., arterioportal (AP) shunt), (5) cyst, and (6) other lesions for which clinical diagnosis was not yet finalized. Here, if more than one lesion was found in the same category, the lesion closest to 1 cm in size was selected. Another radiologist evaluated lesion conspicuity by comparing original and artifact-reduced images for the selected lesions. During evaluation, the radiologist paid special attention on whether the lesion was discernible in both images and whether additional information could be gained after the filter application. Comparison of lesion conspicuity was evaluated side-by-side between original and filtered datasets and rated using a 5-point conspicuity score according to the following scheme: conspicuity was (1) much better in artifact-reduced images compared to original images, (2) better than in artifact-reduced images than original images, (3) the same in both images, (4) better in original than in artifact-reduced images, and (5) much better in original images than in artifact-reduced images.

### MARC filter

**Fig. 2:**
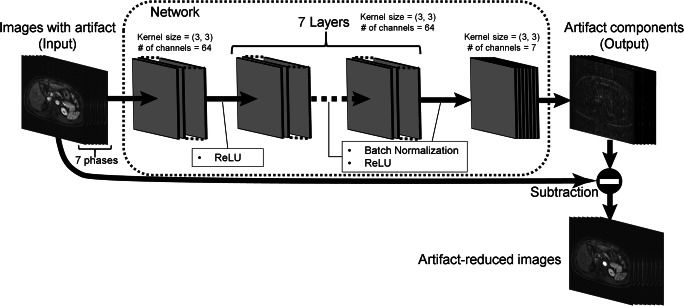
Motion artifact reduction with convolutional network (MARC). The denoising filter, which extracts artifact components from input images, was developed based on a deep convolutional neural network consisting of seven layers

The network, which extracts artifact components from input images, was developed based on a deep convolutional neural network consisting of seven layers [23]. Each layer has two-dimensional convolutions, batch normalizations, and rectified linear units (ReLUs). Seven filters with a kernel size of 3 × 3 were used for the first and the last layers. The remaining layers have sixty-four filters with a kernel size of 3 × 3. We termed this multichannel convolutional neural network–based method MARC (motion artifact reduction with convolutional network) (Fig. 2).

Datasets for the training were generated from the acquired and simulated images of 6 patients, which were not included in the study population. From the acquired datasets, images without motion artifact were selected by a radiologist. Simulation was then performed by adding phase errors to the *k*-space domain, which simulate the rigid motion with periodic and random respiration, as shown in a previous study [23]. Phase error can be expressed as

$$ \phi \left({k}_y\right)=\left\{\begin{array}{c}0,\left|{k}_y\right|<{k}_{y0}\\ {}2\pi \frac{k_y\Delta \mathrm{sin}\left(\alpha {k}_y+\beta \right)}{N},\mathrm{otherwise}\end{array}\right. $$

where *k*_*y*_ is the *k*-space along the phase-encoding direction, Δ denotes the significance of motion, *α* is the period, and *β* is the phase of the sine wave, which determines the frequency and significance of motion.

For the periodic motion, we assumed the frequency and the significance of motion were 0.2–0.7 Hz [24] and 0.0–2.6 cm, respectively. The phase offset of *β* was randomly selected from −*π* to +*π*. To remain contrast information, no phase error was added in the *k*-space center within ±*k*_*y*0_. *k*_*y*0_ of 20 px was used in this study. Random respiration representing the irregular motion was determined as follows: At first, the number of phase encodings, which have phase error, was randomly determined. Then, the significance of the phase error for each phase encoding was also randomly determined line-by-line in the same manner as used for the periodic phase error. The patched images (noisy patch), used for the input of the network, with the size of 96 × 96 × 7, were cropped from the simulated images. The residual patches used for the output layer were cropped from the images which is subtraction of the acquired from simulated images. A total of 14,190 patches with the size of 96 × 96 × 7 were generated. All patches were normalized by dividing them by the maximum value of the artifact images.

### Statistics

Denoising performance was evaluated by comparing motion scores between two groups: (1) original images and (2) the corresponding artifact-reduced images using the Wilcoxon rank sum test with a null hypothesis of equal distributions, where the original images with score 1 and their corresponding artifact-reduced counterparts were excluded. For comparison of lesion conspicuity, the one-tailed, one-sample Wilcoxon signed rank test was used to estimate whether the median score of the sample is less than 3 (no preference between original and artifact-reduced images), which implies a better performance for images after the MARC filter application. A *p* value < 0.05 was considered statistically significant.

To evaluate data quality, interobserver reliability was calculated by using kappa statistics. Hereby, a value below 0.20 defines disagreement, 0.20–0.40 poor agreement, 0.41–0.60 moderate agreement, 0.61–0.80 good agreement, and over 0.80 excellent agreement.

## Results

### Interobserver agreement

Interobserver comparison concerning artifact grading showed good agreement for both original (kappa = 0.79; 95% CI = 0.76, 0.82) and artifact-reduced (kappa = 0.81; 95% CI = 0.78, 0.84) images. Likewise, agreement for lesion conspicuity was good with kappa = 0.61 (95% CI = 0.514, 0.723).

### Motion artifacts in original images

**Fig. 3:**
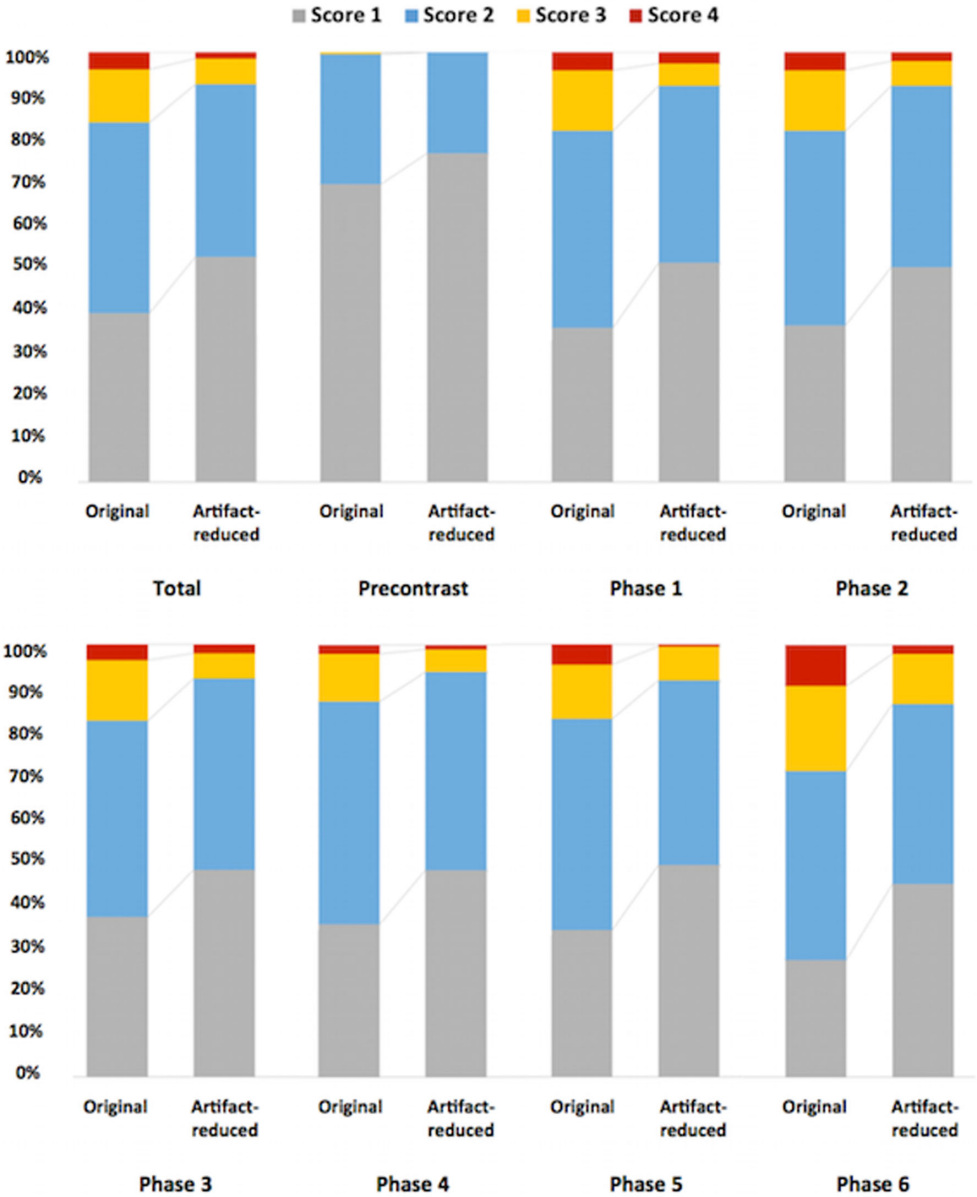
Motion scores before and after MARC application. Over all phases, as well as separate phases before and after artifact reduction

Of the 1344 original image sets, no artifacts were detected in 39.3% (*n* = 528/1344). Minor artifacts were recorded in 44.4% (score 2, *n* = 597/1344), moderate artifacts in 12.3% (score 3, *n* = 165/1344), and severe artifacts in 4.0% (score 4, *n* = 54/1344) (Table 1, Fig. 3). In the image sets of arterial phases only, substantial artifacts (scores 3 and 4) were present in 18.9% (*n* = 218/1152).

**Table 1:** Motion artifact scores for each original image set

	Pre	Art_1	Art_2	Art_3	Art_4	Art_5	Art_6	Total of all phases, *n* (%)	Total of arterial phases, *n* (%)
Motion artifact score									
1	133	69	76	71	68	65	52	528 (39.3)	395 (34.3)
2	58	88	87	87	99	94	84	597 (44.4)	539 (46.8)
3	1	27	27	27	21	24	38	165 (12.3)	164 (14.2)
4	0	0	8	7	4	9	18	54 (4.0)	54 (4.7)
Substantial artifacts									
Scores 3 and 4	1	35	35	34	25	33	56	219 (16.3)	218 (18.9)
Total	192	192	192	192	192	192	192	1344	1152

Pre = pre-contrast phase; Art_1 to Art_6 = arterial phase 1 to 6; motion artifact scores: 1 = no artifacts, 2 = minor artifacts (no effect on diagnostic quality), 3 = distinct artifacts (impeded diagnostic quality), and 4 = severe artifacts (non-diagnostic image quality)

### Performance of artifact reduction

**Fig. 4:**
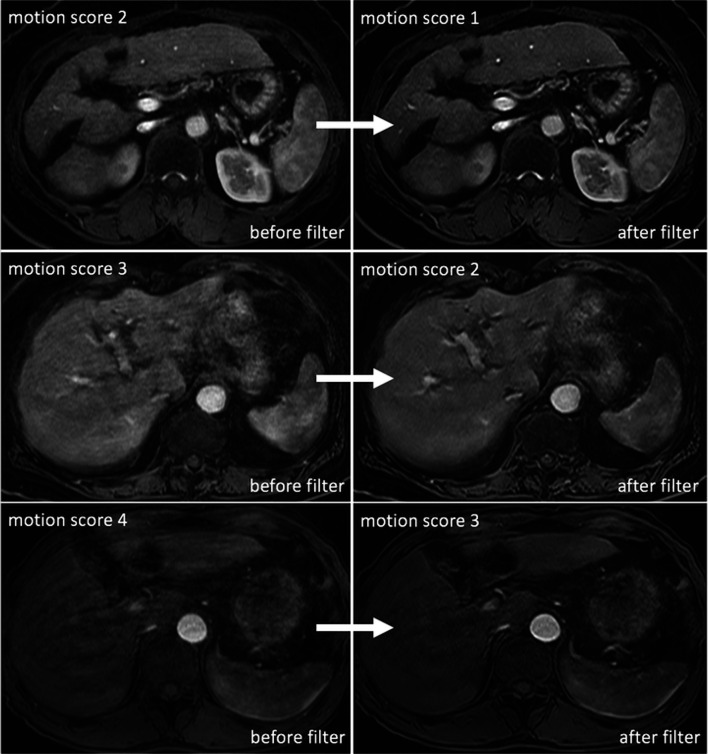
Artifact reduction and improved image quality after filter application. Postprocessing with MARC led to a decrease in motion score from 2 to 1 in 29.9%, from 3 to 2 in 72.1%, and from 4 to 3 in 63.0% of all cases

Image artifact was significantly reduced over all phases from an average motion artifact score (± standard deviation) of 2.53 (± 0.71) in the original MR images to 1.97 (± 0.72) after the MARC filter application (*p* < 0.001). Significant reduction in motion artifacts was shown in artifact-reduced images of each phase (pre-contrast and each arterial phase) (mean grading before vs after filter (± SD)): pre-contrast, 2.02 (± 0.13) vs 1.76 (± 0.43), *p* < 0.001; phase 1, 2.35 (± 0.60) vs 1.93 (± 0.69), *p* < 0.001; phase 2, 2.35 (± 0.60) vs 1.94 (± 0.66); phase 3, 2.34 (± 0.58) vs 1.98 (± 0.63), *p* < 0.001; phase 4, 2.23 (± 0.49) vs 1.92 (± 0.58), *p* < 0.001; phase 5, 2.33 (± 0.60) vs 1.91 (± 0.61), *p* < 0.001; and phase 6, 2.53 (± 0.71) vs 1.97 (± 0.72), *p* < 0.001. Postprocessing with MARC led to a decrease in motion artifact scores from 2 to 1 in 177 of the 597 sets scored 2 in the original images (29.65%), from 3 to 2 in 72.12% (*n* = 119/165), and from 4 to 3 in 62.96% (*n* = 34/54) of all cases (Fig. 4, Table 1).

### Lesion conspicuity

**Fig. 5:**
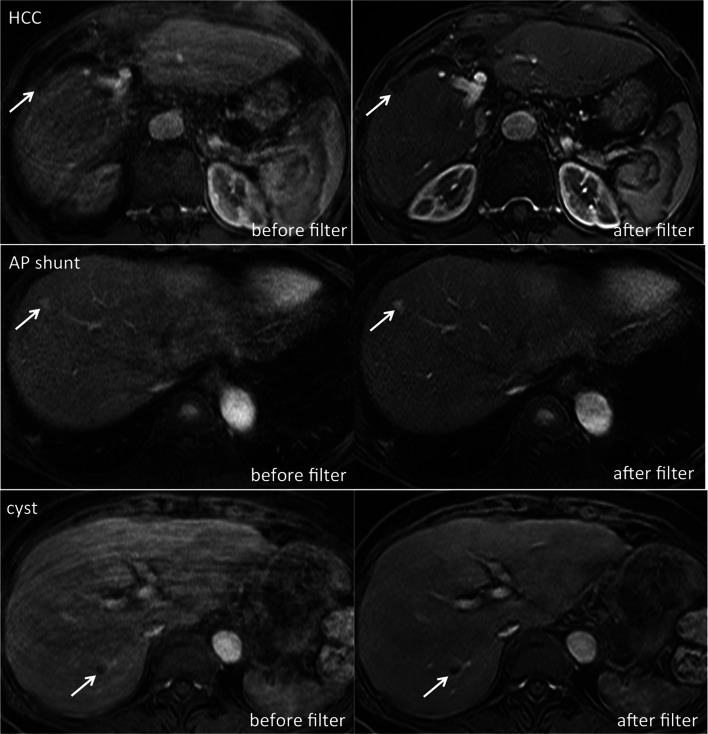
Lesion conspicuity before and after MARC application. Axial T1-weighted MR images in patients diagnosed with HCC, AP shunt and liver cyst (lesions are each indicated by arrow) show improved lesion conspicuity following filter application compared to the original images (conspicuity scores of 1, 2, and 1, respectively). Diagnosis of the liver cyst and AP shunt is greatly improved after MARC application, and identification of the HCC lesion, which could have easily be mist on the original images, is greatly improved

In the total study population, 159 focal liver lesions were chosen in 159 subjects (110 male, 49 female, mean age 68.8 ± 10.1 years): malignant tumors (*n* = 56; 48 HCCs, 8 metastases), benign tumors (*n* = 10; 8 hemangiomas, 2 focal nodular hyperplasia (FNHs)), benign non-tumorous lesions (*n* = 46; 45 AP shunts, 1 inflammatory lesion), cysts (*n* = 44), and other lesions (*n* = 3; 1 unspecified hypervascular lesion, 1 hyperplastic nodule, 1 unknown ring-enhancing lesion). Mean lesion size was 9.00 mm (range 2.7–44.2 mm). After the filter application, all reference lesions were still discernable. Side-by-side comparison of lesion conspicuity in original and artifact-reduced images showed that lesions were more conspicuous after MARC application (*p* < 0.001). Reader 1 rated artifact-reduced images much better against the originals (conspicuity score 1) in 30 and better (score 2) in 75 lesions (Fig. 5). No preference (score 3) was declared in 52 subjects. Preference for original images (score 4) was recorded in only 2 lesions. No lesion was scored as 5. The second reader rated conspicuity score 1 in 27 cases, score 2 in 58, score 3 in 68, and score 4 in 6 cases. Likewise, no lesion was scored as 5.

## Discussion

With this retrospective analysis, we revealed that postprocessing using the MARC filter improved image quality in terms of respiratory motion artifacts in multiple arterial phase image acquisition with gadoxetate disodium.

Respiratory motion artifacts are a major problem in MR imaging of the abdomen. As described in previous studies, gadoxetate disodium–related transient severe motion degrades the quality of arterial phase images [1–4, 14], resulting in decreased diagnostic accuracy, particularly for the detection and characterization of focal hepatic lesions [6, 7]. Among the multitude of strategies proposed, especially fast scanning techniques using compressed sensing offer a simple approach to minimize motion artifacts [11, 12]. Pietryga et al [2] showed that the use of multiple arterial phase acquisition within a rapid single breath-hold provides adequate well-timed late hepatic arterial phase images in most patients with TSM. In general, multiple arterial phase acquisition with view sharing technique can improve the time resolution. However, it often requires longer time for breath-hold. In our study, the acquisition time was relatively long, which probably led to more artifacts in the last 1–2 phases. In the meantime, most cases had phases without artifact in the front half, where appropriate late arterial phase images were included. These images without artifact can be used as a reference of anatomical details when convolutional neural network (CNN) works for artifact reduction. Taking these findings into account, in our study, we developed a deep learning filter based on CNN, which works in combination with multi-arterial phase acquisition using DISCO to achieve motion artifact removal in MRI of the liver.

Deep learning methods recently have shown promising performances on various vision tasks, which also include fields of importance for medical imaging. Deep learning is based on a neural network with a high number of hidden layers [25]. Central among the multitude of existing approaches, the feedforward technique of CNN shows emerging applications in image reconstruction, lesion/artifact detection, denoising, segmentation, or super resolution [19, 26–28]. CNN employs trainable filters and convolution operations between input and output filters, reducing the amount of parameters necessary to be learned compared to classic multilayer perception methods [29, 30]. Few feasibility studies reported on motion artifact reduction. For instance, Hauptmann et al [20] generated a CNN for the suppression of spatiotemporal artifacts on cine images of cardiac MR studies. Pawar et al [22] successfully applied an encoder-decoder convolutional neural network on clinical brain scans for motion correction. However, clinically validated studies, especially focusing on motion artifacts in abdominal MR scans, remain scarce. In our study, the newly developed CNN filter MARC significantly improved image quality by reducing motion artifacts in dynamic liver MRI. We were able to apply this post hoc motion correction in a clinical setting. Furthermore, in almost all cases, observers preferred artifact-reduced MR images for evaluating focal liver lesions compared to the original ones or rated both datasets as equal. Comparing original and artifact-reduced images side-by-side, no loss of anatomical or pathological information after MARC application could be detected.

In our opinion, the use of the MARC filter offers a new perspective in artifact-reduced high-quality MRI. The application of MARC in combination with multi-arterial phase imaging technique is able to reduce artifacts in liver MRI. This can be of value, for instance, in cases where pseudolesions occur in original images due to artifacts, which might be clarified after artifact removal. The impact of artifact reduction in other regions, e.g., MRI of the lung, pancreas, kidney, and bowel, needs to be further explored. Nevertheless, an improvement in image quality does not automatically result in a better diagnosis.

Apart from that, advanced deep learning techniques, such as generative adversarial networks (GANs) [31], could improve our approach. Jiang et al [32] proposed a respiratory motion correction method for abdominal imaging using U-Net and GAN. Armanious et al [33] demonstrated that GAN enables rigid and non-rigid motion correction. These results show GAN as a promising approach for motion artifact correction, although there is a difficulty in determining the structure and hyperparameters due to simultaneous training of two networks of a generator and a discriminator [34].

Our study has several limitations. First, the study design was retrospective. Since the observers were informed about the study purpose, they might have been sensitized to TSM occurrence while examining MR images. This, however, accounts for original and artifact-reduced datasets alike. Furthermore, the introduced filter trained with the simulated datasets was not generalized for all possible cases since the simulation was performed based on the limited condition of respiratory motion. Likewise, the input of MARC filter was multiple arterial phase images acquired with a specific MRI pulse sequence/view sharing acquisition technique (DISCO). Additional validation in terms of diagnostic ability, i.e., sensitivity, specificity, and accuracy, with a larger patient cohort or different MRI sequence parameters might be beneficial for further improvement of the developed filter algorithm. There is still room for further improvement of motion artifact simulation. Since DISCO sequence uses a complicated scheme consisting of ARC reconstruction and irregular sub-sampling *k*-space ordering from low- to high-frequency components, phase corruption due to motion could lead to aliasing artifact in addition to motion artifact. Since the training datasets were generated from DICOM images in this study, it is difficult to simulate these complicated mechanisms. Therefore, we implemented the approximated simulation with straightforward centric *k*-space order. Further consideration will be needed to improve de-aliasing performance by generating datasets from complex raw data with exact acquisition ordering. Because the proposed CNN-based approach removes motion artifact by extracting deep features that are optimized with the training process, inappropriate training such as inadequate network structure and insufficient size of training datasets degrades the processing performance of the network. Therefore, a larger volume of training datasets or adopting a sophisticated network could yield further improvement.

In conclusion, the clinical evaluation of our newly developed CNN-based filter MARC significantly improved image quality of gadoxetate-enhanced MRI of the liver by reducing motion artifacts, especially in cases with substantial artifacts. This approach is of high clinical value in subjects who failed breath-hold in the scan.

## Abbreviations

AP: Arterioportal
CNN: Convolutional neural network
FNH: Focal nodular hyperplasia
HCC: Hepatocellular carcinoma
MARC: Motion artifact reduction with convolutional neural network
MRI: Magnetic resonance imaging
ReLU: Rectified linear unit
TSM: Transient severe motion
